# Multi-omics analysis of diabetic pig lungs reveals molecular derangements underlying pulmonary complications of diabetes mellitus

**DOI:** 10.1242/dmm.050650

**Published:** 2024-07-23

**Authors:** Bachuki Shashikadze, Florian Flenkenthaler, Elisabeth Kemter, Sophie Franzmeier, Jan B. Stöckl, Mark Haid, Fabien Riols, Michael Rothe, Lisa Pichl, Simone Renner, Andreas Blutke, Eckhard Wolf, Thomas Fröhlich

**Affiliations:** ^1^ Laboratory for Functional Genome Analysis (LAFUGA), Gene Center, LMU Munich, 81377 Munich, Germany; ^2^ German Center for Diabetes Research (DZD), 85764 Neuherberg, Germany; ^3^ Chair for Molecular Animal Breeding and Biotechnology, Gene Center and Department of Veterinary Sciences, LMU Munich, 81377 Munich, Germany; ^4^ Center for Innovative Medical Models (CiMM), LMU Munich, 85764 Oberschleißheim, Germany; ^5^ Institute for Veterinary Pathology, Center for Clinical Veterinary Medicine, LMU Munich, 80539, Germany; ^6^ Metabolomics and Proteomics Core (MPC), Helmholtz Munich, 85764 Neuherberg, Germany; ^7^ Lipidomix GmbH, 13125 Berlin, Germany

**Keywords:** Diabetes, Biobank, Insulin deficiency, Lipidome, Lipoxygenase, Lung, Pig model, Proteome

## Abstract

Growing evidence shows that the lung is an organ prone to injury by diabetes mellitus. However, the molecular mechanisms of these pulmonary complications have not yet been characterized comprehensively. To systematically study the effects of insulin deficiency and hyperglycaemia on the lung, we combined proteomics and lipidomics with quantitative histomorphological analyses to compare lung tissue samples from a clinically relevant pig model for mutant *INS* gene-induced diabetes of youth (MIDY) with samples from wild-type littermate controls. Among others, the level of pulmonary surfactant-associated protein A (SFTPA1), a biomarker of lung injury, was moderately elevated. Furthermore, key proteins related to humoral immune response and extracellular matrix organization were significantly altered in abundance. Importantly, a lipoxygenase pathway was dysregulated as indicated by 2.5-fold reduction of polyunsaturated fatty acid lipoxygenase ALOX15 levels, associated with corresponding changes in the levels of lipids influenced by this enzyme. Our multi-omics study points to an involvement of reduced ALOX15 levels and an associated lack of eicosanoid switching as mechanisms contributing to a proinflammatory milieu in the lungs of subjects with diabetes mellitus.

## Teaser

**Summary:** First multi-omics characterization of lung tissue in a clinically relevant pig model of insulin-deficient diabetes showed pronounced derangements in proteomic and metabolomic profiles, pointing to pulmonary complications.

## INTRODUCTION

Diabetes mellitus alongside its associated complications has emerged as a global health problem, the prevalence of which has increased over the past decades. Diabetes causes profound long-term molecular effects on multiple tissues and organs. Traditionally, the chronic complications of diabetes are classified as macrovascular and microvascular complications (Vithian and Hurel, 2010; Hinkel et al., 2017; Kleinwort et al., 2017). The rich vascularization of the lungs and the abundance of connective tissue suggests a susceptibility to diabetic microvascular damage (Mameli et al., 2021). The pathophysiology of pulmonary symptoms in diabetes is complex and thus far not fully understood. In addition, pulmonary damage is mostly subclinical and therefore difficult to detect (Mameli et al., 2021; Hsia and Raskin, 2007). Multiple studies have pointed to various pulmonary complications in diabetes (reviewed by Mameli et al., 2021; Kolahian et al., 2019; Rajasurya et al., 2020). In particular, an increased susceptibility to respiratory infections is frequently observed in patients with diabetes. As the respiratory tract is constantly exposed to pathogens, defence mechanisms in the lung are crucial. Higher hospitalization and mortality rates were observed in patients with diabetes with viral or bacterial infections such as influenza (Klekotka et al., 2015) and COVID-19 (Lim et al., 2021). Additionally, diabetes significantly increases mortality rates in patients with idiopathic pulmonary fibrosis (Hyldgaard et al., 2014). Furthermore, individuals with diabetes are at increased risk to develop further pulmonary conditions such as asthma, pulmonary fibrosis and chronic obstructive pulmonary disease (COPD) (Ehrlich et al., 2010).

Thus far, the research has been mainly focused on epidemiological associations between diabetes and impaired lung function. However, for prevention and intervention strategies, it is crucial to understand underlying molecular mechanisms. Several rodent models have been established and provided valuable insights into the onset and progression of diabetes (King, 2012). Streptozotocin (STZ)-induced β-cell injury in rodents is commonly used as a model of type 1 diabetes (Furman, 2021). However, the confounding effects of STZ, especially on the immune system (Muller et al., 2011), complicate the interpretation of the findings. Furthermore, rodent models frequently display lower clinical relevance due to fundamental physiological differences from humans. In this context, porcine models, which better reflect the human system, are becoming increasingly important in diabetes research to bridge the gap between proof-of-concept studies in rodents and clinical trials (Renner et al., 2020). The pig is a valuable model in the context of respiratory medicine, as porcine and human lungs share many anatomical, histological, biochemical and physiological characteristics (Judge et al., 2014). Furthermore, functional similarities of the porcine host defence proteins with their human counterparts make the pig an excellent model to study the pathogenesis of respiratory inflammation (Rogers et al., 2008). We thus investigated lung samples from *INS*^C94Y^ transgenic pigs, a tailored large animal model for mutant *INS* gene-induced diabetes of youth (MIDY), characterized by impaired insulin secretion, β-cell loss, and chronic hyperglycaemia. MIDY pigs exhibit a stable diabetic phenotype without further manipulation because of a clinically relevant impairment of β-cells (Renner et al., 2013). Furthermore, MIDY pigs develop diabetes-related alterations in various tissues including the myocardium (Hinkel et al., 2017), retina (Kleinwort et al., 2017), immune cells (Giese et al., 2020), liver (Backman et al., 2019) and adipose tissue (Renner et al., 2020; Flenkenthaler et al., 2021).

In the present study, proteomics and targeted analysis of relevant lipid molecules were performed on lung tissue samples from the Munich MIDY pig biobank (Blutke et al., 2017a) to systematically address pulmonary changes in response to chronic insulin deficiency and hyperglycaemia. Additional immunohistochemical and quantitative morphological analyses were carried out to localize differentially abundant key molecules in their pathophysiological context.

## RESULTS

### Overview of proteome differences

**Fig. 1.**
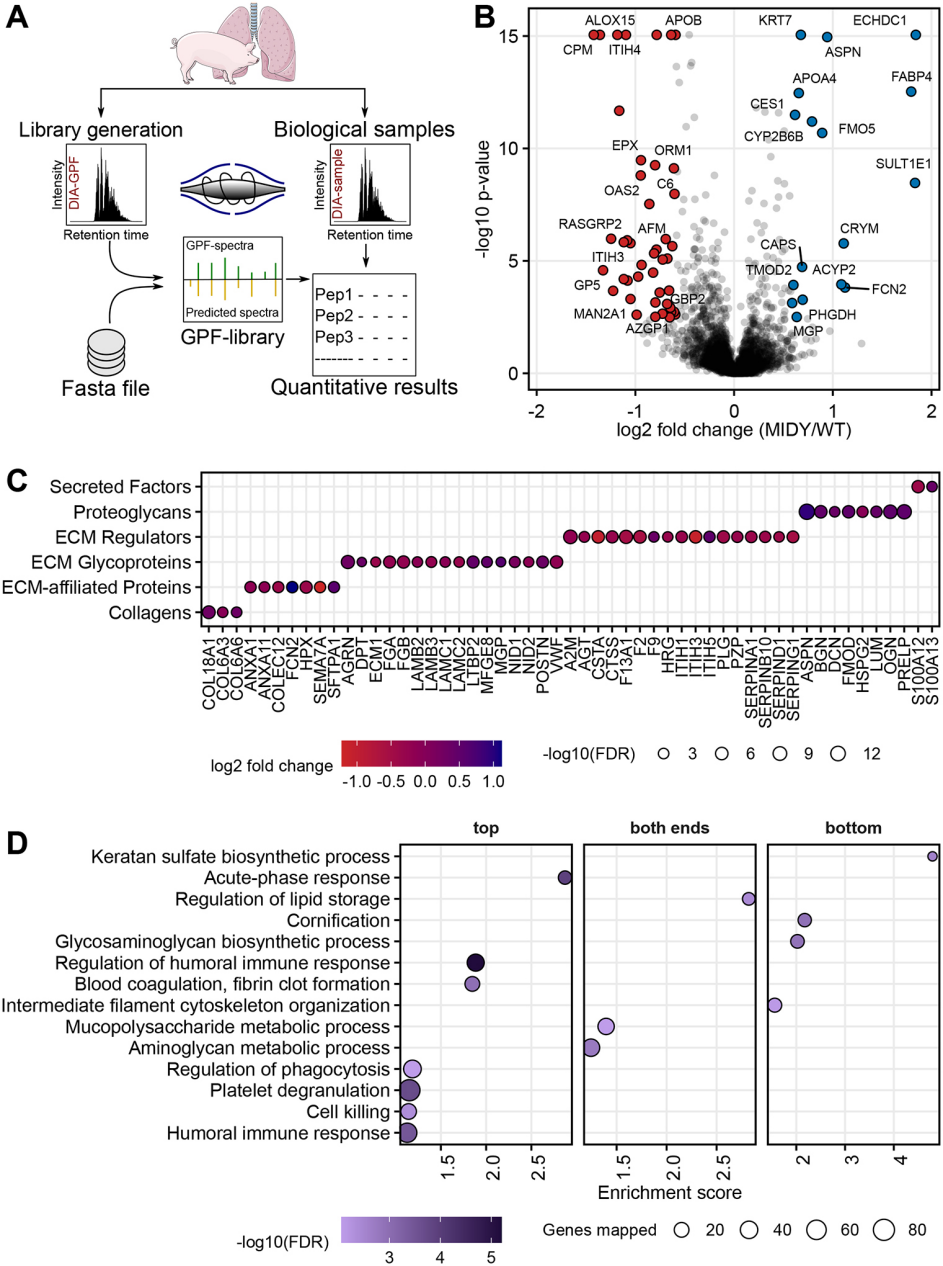
**Quantitative proteome analysis of lung tissue from WT and MIDY pigs.** (A) Experimental design. Lung tissue proteomes from the MIDY and WT animals were analysed using a multi-injection gas-phase fractionation (GPF) data-independent acquisition (DIA) as described previously (Pino et al., 2020; Demichev et al., 2020). (B) Volcano plot visualization of proteome abundance changes between MIDY and WT samples. Protein abundance changes with Benjamini–Hochberg-corrected *P*-value ≤0.05 and fold change ≥1.50 in the MIDY lung are coloured in red and blue for downregulation and upregulation, respectively. (C) Abundance change of proteins that are part of the extracellular matrix according to Naba et al. (2012). The colours of the circles correspond to the log_2_(fold change) of proteins (red indicates downregulation and blue indicates upregulation) and the sizes of the circles indicate the significance of the protein abundance change. FDR, false discovery rate. (D) Pre-ranked enrichment analysis using STRING with gene sets according to Gene Ontology (GO) biological process databases was used to reveal processes enriched in the top (downregulated) or bottom (upregulated) of a ranked list of genes. Significantly enriched GO biological processes (FDR<0.05) were summarized with REVIGO by grouping semantically similar ontology terms. Processes related to downregulated proteins (left column), upregulated proteins (right column) and simultaneously related to more and less abundant proteins (middle column) are shown. The sizes of the circles indicate the corresponding numbers of the quantified proteins (referred genes mapped in the figure) associated with the pathway, and colours indicate the significance of enrichment. Fold enrichment represents the magnitude of overrepresentation.

To investigate the molecular effects of chronic insulin deficiency and hyperglycaemia on the lung tissue proteome, we performed a label-free liquid chromatography (LC)-tandem mass spectrometry (MS/MS) analysis of lung samples from MIDY and wild-type (WT) animals. Using data-independent acquisition (DIA) (Fig. 1A), we identified 45,411 distinct peptides from 5465 protein groups with high confidence (false discovery rate <0.01) (Tables S1 and S2). The dataset has been submitted to the ProteomeXchange Consortium via the PRIDE partner repository (PXD038014). Quantitative analysis using the MS-EmpiRe workflow (Ammar et al., 2019) detected 265 proteins changed in abundance between MIDY and WT samples with Benjamini–Hochberg-corrected *P*-value ≤0.05 (Table S3), out of which 61 proteins were changed in abundance by at least 1.5-fold (Fig. 1B).

The protein with the highest abundance increase (3.6-fold) in MIDY lungs was ethylmalonyl-CoA decarboxylase 1 (ECHDC1). Likewise, the levels of other proteins involved in lipid catabolic processes, such as oestrogen sulfotransferase (SULT1E1), fatty acid-binding protein 4 (FABP4), apolipoprotein A4 (APOA4) and carboxylic ester hydrolase (CES1), were elevated. Furthermore, members of the small leucine-rich proteoglycan (SLRP) family were more abundant in MIDY versus WT samples. The most prominent was asporin (ASPN) with a 1.9-fold increase. Additionally, correlation analysis revealed a significant correlation between the levels of SLRP proteins (Fig. S1). Pulmonary surfactant-associated protein A (SFTPA1) was also elevated in MIDY lungs, albeit the abundance change was moderate (Benjamini–Hochberg-corrected *P*-value=0.005, ∼1.5-fold increase).

Among the most prominently reduced proteins in MIDY lungs were carboxypeptidase M (CPM, 2.7-fold decrease) and polyunsaturated fatty acid (PUFA) lipoxygenase ALOX15 (ALOX15, 2.6-fold decrease). Several members of the complement and coagulation cascades were also reduced, of which complement component C6 (C6) was the most pronounced with a 1.8-fold decrease. A large fraction of differentially abundant proteins in MIDY compared to WT pigs were extracellular matrix (ECM) proteins. We classified these proteins into the following groups: secreted factors, proteoglycans, ECM regulators, ECM glycoproteins, ECM-affiliated proteins and collagens (Fig. 1C). Similarly, proteins involved in the biological processes and pathways related to insulin homeostasis are visualized in Fig. S2.

Furthermore, to functionally characterize proteome alterations between MIDY and WT lungs, a pre-ranked gene set enrichment analysis using STRING was performed. The detailed results of the enrichment analysis are provided in Table S4 and are visualized in Fig. 1D. Gene sets, such as ‘acute-phase response’, ‘regulation of humoral immune response’, ‘blood coagulation’, ‘regulation of phagocytosis’, ‘platelet degranulation’, ‘cell killing’ and ‘humoral immune response’, were enriched among the proteins decreased in abundance, whereas proteins related to ‘keratan sulphate biosynthetic process’, ‘cornification’, ‘glycosaminoglycan biosynthetic process’ and ‘intermediate filament cytoskeleton organization’ were enriched among the upregulated proteins. An enrichment of proteins related to ‘lipid storage’, ‘mucopolysaccharide metabolic process’ and ‘aminoglycan metabolic process’ was simultaneously found in the sets of more and less abundant proteins.

### Protein localization studies and quantitative stereology

**Fig. 2.**
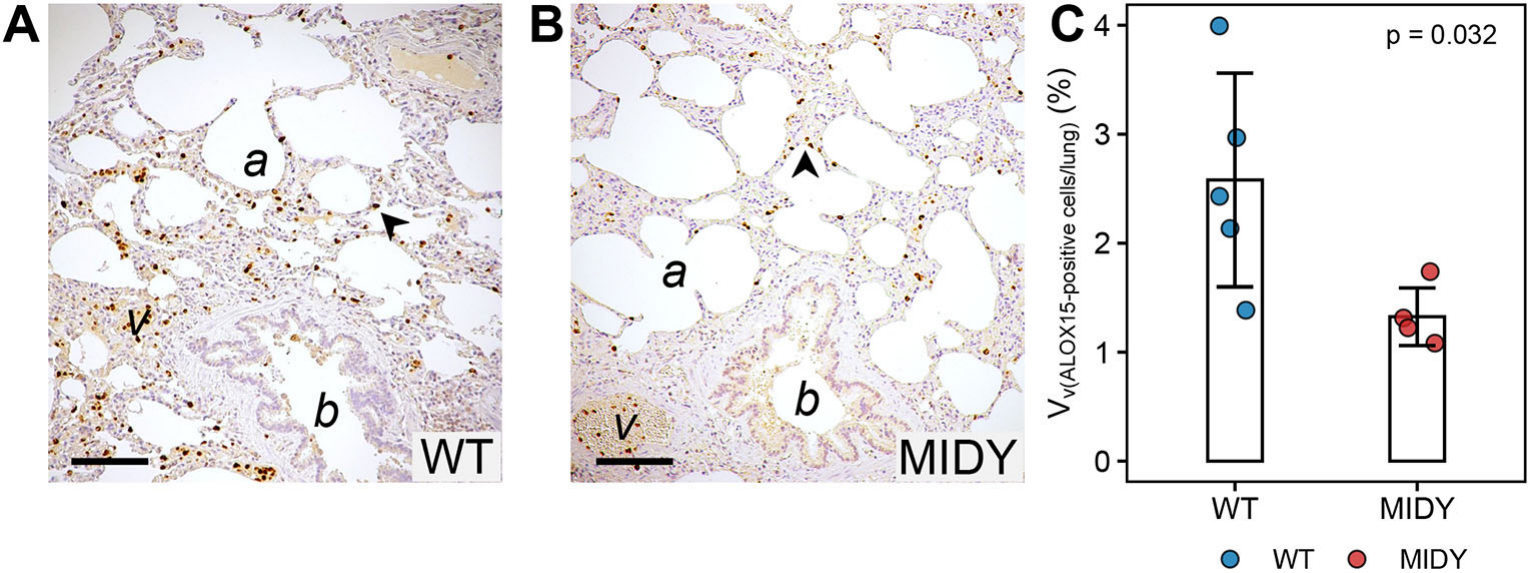
**Analysis of ALOX15 in lung sections of WT and MIDY pigs.** (A,B) Immunohistochemical detection of ALOX15 in paraffin lung sections of WT (A) and MIDY pigs (B). Histological landmarks [alveoli (a), blood vessels (v) and bronchioli (b)] are indicated. ALOX15-positive cells (dark brown) are present within alveolar septae (arrowheads) and inside vascular lumina. Chromogen: DAB; nuclear counterstain: haemalum. Scale bars: 100 µm. (C) Volume densities of ALOX15-positive cells within the lung of WT and MIDY pigs. Statistical significance of the difference was assessed using the Mann–Whitney *U*-test. The bar diagrams show means and standard deviations.

In lung tissue sections of MIDY and WT pigs, ALOX15 immunoreactivity was present in mononuclear cells within alveolar walls and inside the vascular lumina (Fig. 2A,B). Confirming the significantly reduced ALOX15 protein levels in the MIDY lung tissue identified by proteomic analysis, quantitative stereological analysis revealed a significantly decreased volume density of ALOX15-positive cells within the lung tissue (Fig. 2C). The volume density of interstitial connective tissue in the lung tissue (excluding air-filled spaces) of MIDY pigs was slightly but not significantly increased (*P*=0.19) compared to that of WT animals (Fig. S3).

### Overview of lipidome differences

**Fig. 3.**
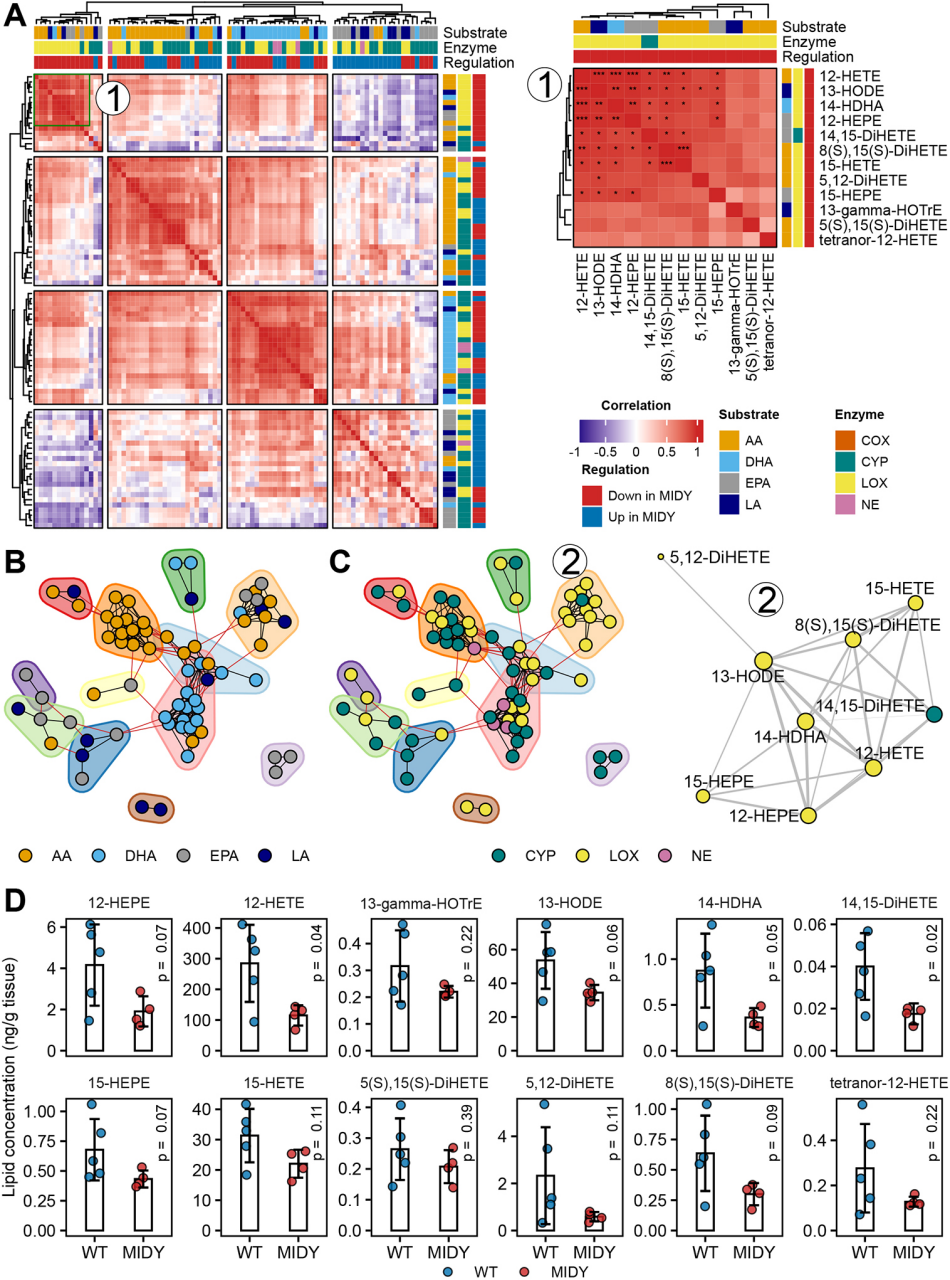
**Correlation analysis of eicosanoid levels from WT and MIDY lungs.** (A) Global correlation map of eicosanoid levels on the left with an inset of the selected cluster (1) on the right. The correlation was estimated using the non-parametric Spearman rank correlation coefficient. Red and blue patches in the correlation map indicate positive and negative correlations, respectively. Columns and rows of the heatmap are annotated for each lipid, based on the substrates and enzymes involved in their production. The regulation column indicates the abundance change of eicosanoids in MIDY versus WT lungs. The correlation map was partitioned into homogenous regions using the k-means method (k=4). The correlation map on the right is labelled with an asterisk according to the significance (*P*-value) of the correlation after multiple testing correction for all pairwise comparisons using the Benjamini–Hochberg method. *P<0.05; **P<0.01; ***P<0.001. (B,C) Correlation between eicosanoid levels shown as a network. Each node corresponds to a single lipid and edges are drawn between highly correlated (|ρ|>0.8) molecules. Nodes with dense connections were grouped using the random walk-based community detection algorithm (coloured drawings around the group of nodes). The networks with nodes are coloured based on a substrate (B) and enzyme (C), with an inset of the selected community network (2) (right) that was filtered for the significant correlations (Benjamini–Hochberg-corrected *P*-value <0.05). The edge thickness in the right cluster (2) corresponds to the magnitude of the correlation (ρ) and the size of the node to the number of its adjacent edges. AA, arachidonic acid; COX, cyclooxygenase; CYP, cytochrome P450; DHA, docosahexaenoic acid; EPA, eicosapentaenoic acid; LA, linoleic acid; LOX, lipoxygenase; NE, non-enzymatic. (D) Eicosanoid levels from the selected clusters (cluster 1 in panel A, cluster 2 in panel B) in MIDY versus WT lungs. Statistical significance of the difference was assessed using two-tailed unpaired Welch's *t*-test. The bar diagrams show means and standard deviations.

**Fig. 4.**
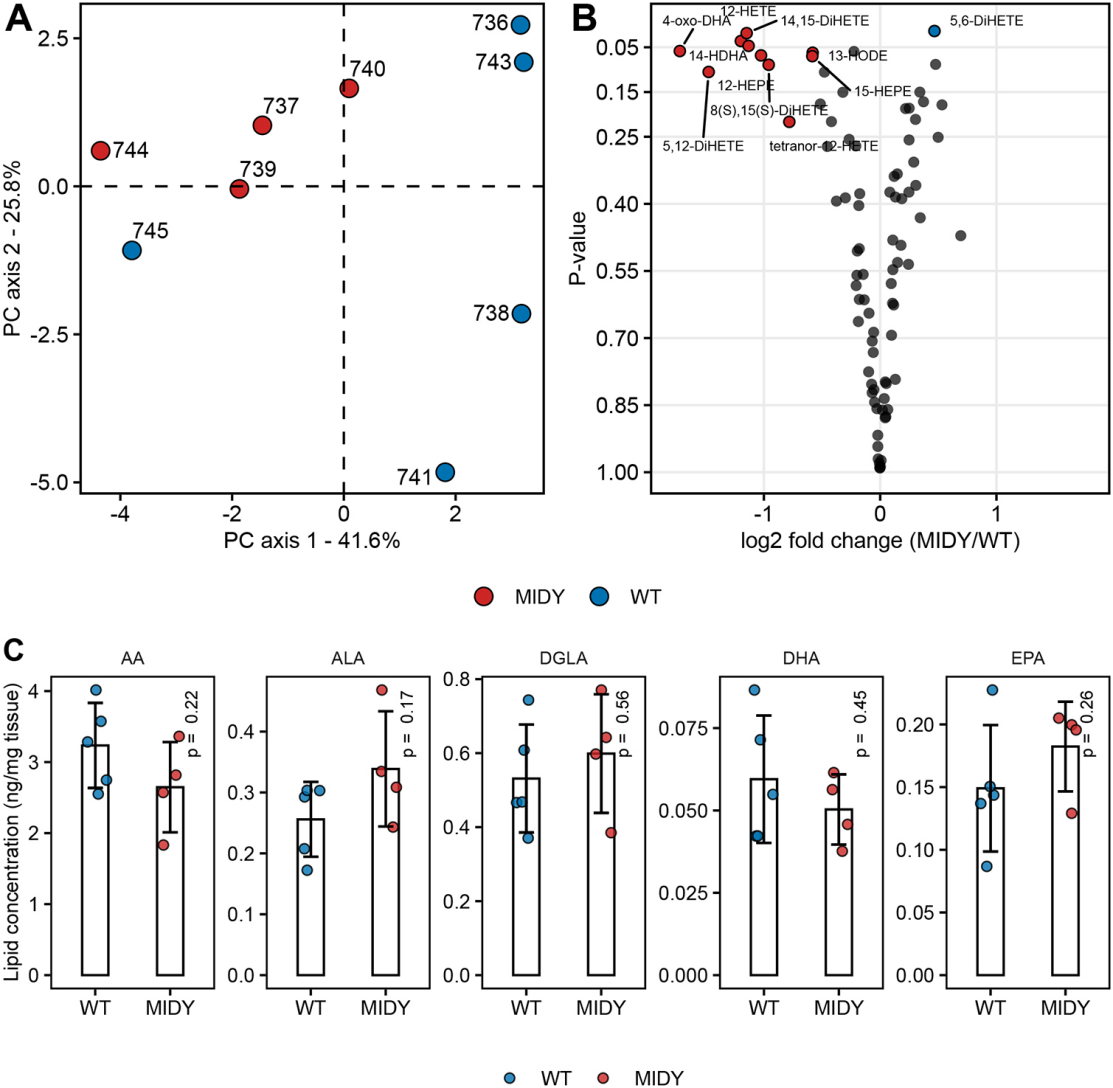
**Eicosanoid levels in lung tissue from WT and MIDY pigs.** (A) Unsupervised principal component analysis (PCA) based on log-transformed lipid levels from MIDY (animal identifiers: 737, 739, 740 and 744) and WT (animal identifiers: 736, 738, 741, 743 and 745) animals. The first two principal components (PCs) explained 67.4% of the total variance. (B) Volcano plot of quantified eicosanoid levels obtained from the univariate statistics showing log_2_(fold change) and *P*-values determined by two-tailed unpaired Welch's *t*-test. (C) Polyunsaturated fatty acid precursor levels in a free state from MIDY and WT lungs. Statistical significance of the difference was assessed using two-tailed unpaired Welch's *t*-test. The bar diagrams show means and standard deviations. AA, arachidonic acid; ALA, α-linolenic acid; DGLA, dihomo-γ-linolenic acid; DHA, docosahexaenoic acid; EPA, eicosapentaenoic acid.

To clarify if the markedly reduced levels of ALOX15 in the MIDY animals affect the total level of eicosanoids, we used mass spectrometry-based targeted lipidomics and compared eicosanoid levels from MIDY and WT lungs. The results are shown in Table S5. A global correlation map of all quantified eicosanoids is shown in Fig. 3A and Table S6. Hierarchical clustering revealed several clusters of molecules that share the same biosynthetic pathway and show a similar regulation trend across animals. Hierarchical clustering revealed four homogenous regions, of which one, consisting of lipids produced mainly by a lipoxygenase (LOX) pathway, was particularly interesting. Magnification of this cluster (Fig. 3A, right inset) shows a heatmap of lipids with strong correlation to each other, and some of these correlations remained significant after adjusting for all pairwise comparisons using the Benjamini–Hochberg method. Focusing on the hypothesis of eicosanoid co-regulation in the MIDY lung, we visualized highly correlated (|ρ|>0.8) lipids as a network (Fig. 3B,C). The community detection algorithm revealed several densely populated subnetworks. To visualize whether distinct communities contain lipids that share the same biosynthetic pathway, we coloured the nodes according to the substrate (Fig. 3B) and enzyme (Fig. 3C). In agreement with Fig. 3A, dense clusters with strong associations across biomolecule classes were apparent. Fig. 3C further shows a network for the selected community with significantly (ρ>0.8 and Benjamini–Hochberg-corrected *P*-value <0.05) correlated lipids. Fig. 3D shows the trend of reduced eicosanoid levels in MIDY compared to WT lungs from the selected cluster (cluster 1 in Fig. 3A, cluster 2 in Fig. 3C). Next, principal component analysis (PCA) was performed on the entire dataset (Fig. 4A), which showed moderate separation between samples from MIDY and WT animals. A volcano plot shows the abundance change of each quantified eicosanoid (Fig. 4B). Furthermore, some of the PUFA precursors in a free state were quantified (Fig. 4C; Table S8).

### Multi-omics data integration

**Fig. 5.**
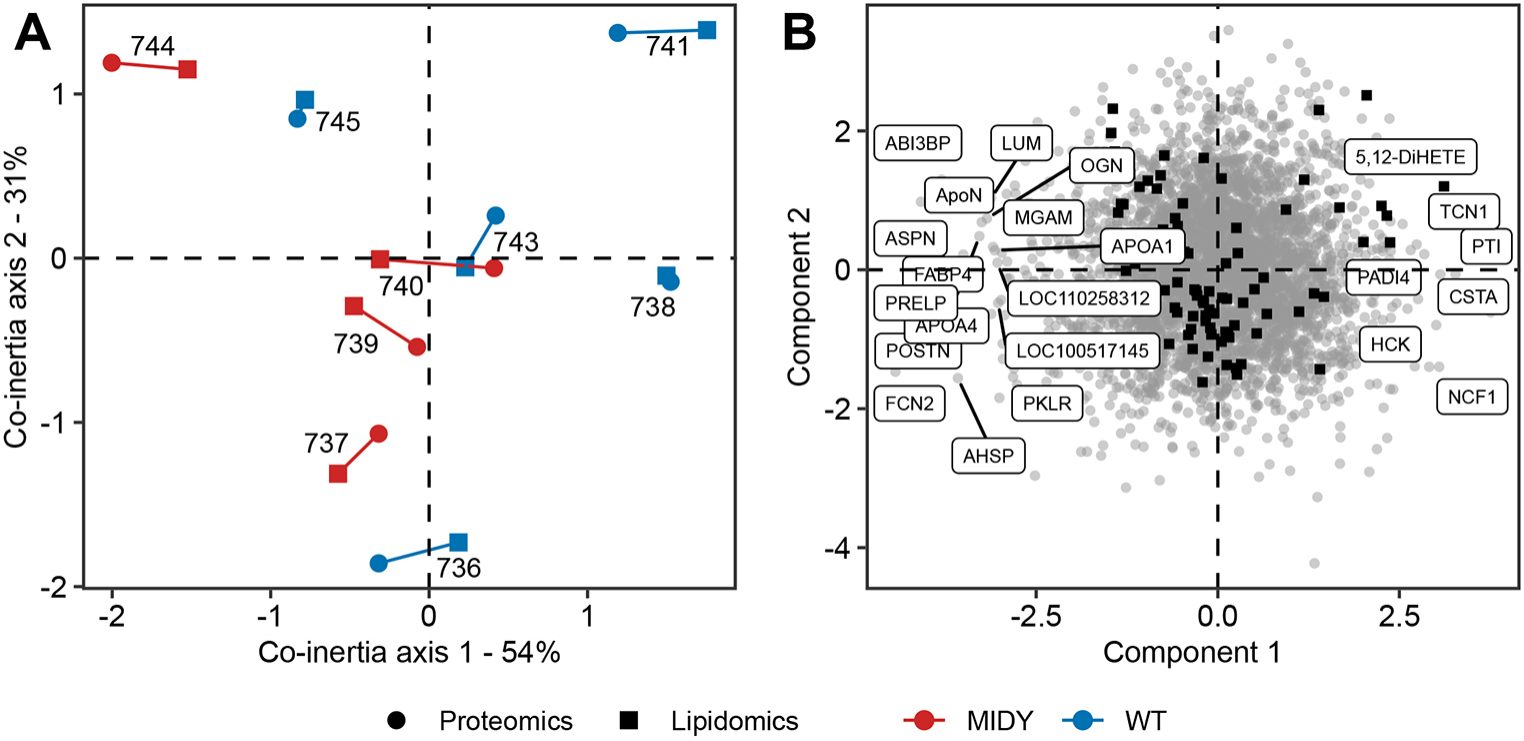
**Omics data integration.** (A,B) Multiple co-inertia analysis of lipidome and proteome data from the MIDY (animal identifiers: 737, 739, 740 and 744) and WT (animal identifiers: 736, 738, 741, 743 and 745) lungs showing the first two components in the sample (A) and variable (B) space. Circles and squares represent the proteome and lipidome data of a given animal, respectively. Short lines in the sample space (A) indicate a higher cross-omics correlation. The RV coefficient (RV=0.78, 500 permutations, *P*=0.04) shows the correlation of two datasets. An RV value close to 1 indicates a strong correlation. Proteins and lipids with high scores in component 1 and component 2 are labelled in a variable space (B).

For multi-omics data integration, co-inertia analysis (CIA) (Meng et al., 2014) was used. Graphical representation of samples (Fig. 5A) and variables (Fig. 5B) on a lower-dimensional space allows interpretation of global variance structure and identification of the most informative biomolecules across datasets. CIA of the proteome (circle) and lipidome (square) revealed a significant relationship (RV coefficient=0.78, 500 permutations, *P*=0.041). The corresponding score plot shows the proteins and lipids responsible for partitioning MIDY and WT samples on the CIA plot. Although it does not display clear clusters, the CIA showed trends towards separation of MIDY and WT samples.

## DISCUSSION

To reveal biological processes and pathways altered by insulin deficiency in the lung and to identify molecular key drivers of these alterations, a multi-omics analysis combining in-depth DIA proteomics, and quantitative readouts of relevant lipid molecules was performed.

The crucial lipid–protein mixture that reduces alveolar surface tension and facilitates breathing is the pulmonary surfactant (Milad and Morissette, 2021), which covers the entire alveolar surface of the lungs (Zuo et al., 2008). Defects in the stimulation of pulmonary surfactant production have been observed in various medical conditions such as COPD (Obeidat et al., 2017) and idiopathic pulmonary fibrosis (Beike et al., 2019). These defects might also be contributing factors to airway dysfunction in diabetes (Foster et al., 2010). Surfactant proteins leak from the alveolar space into the bloodstream, when the alveolar–capillary barrier is damaged, which makes them useful biomarkers for lung injury (López-Cano et al., 2022). We detected a ∼50% increase of the pulmonary surfactant-associated protein A (SFTPA1, also known as SP-A) in MIDY compared to WT pig lungs (Benjamini–Hochberg-adjusted *P*-value <0.005). SP-A is the major protein component of the surfactant and regulates surfactant phospholipid synthesis, secretion and recycling (Khubchandani and Snyder, 2001). Insulin is known to inhibit expression of SP-A in the lung (Rucka et al., 2013; Miakotina et al., 2002); therefore, increased abundance of SP-A in our study is in line with insulin deficiency in MIDY pigs. The clinical relevance of our finding is supported by a randomized population-based study revealing elevated circulating SP-A levels in the blood of patients with glucose intolerance and diabetes (Fernández-Real et al., 2008). SP-A levels were also elevated in the lung of obese diabetic rats compared to lean nondiabetic controls (Foster et al., 2010). The observed increased abundance of SP-A in the MIDY model may reflect the diabetes-associated impairment of pulmonary diffusing capacity reported in children and adolescents with type 1 diabetes (Mameli et al., 2021).

Besides pulmonary surfactant, the composition and function of lung ECM also become markedly deranged due to pathological tissue remodelling in diabetes mellitus (Zhou et al., 2018). Excessive production of ECM components and nonenzymatic glycation of ECM proteins due to hyperglycaemia lead to matrix stiffening, remodelling the lung tissue structure and promoting pulmonary fibrosis. Secreted factors such as transforming growth factor β1 (TGFB1) and connective tissue growth factor (CTGF, also known as CCN2) are the notorious pro-fibrotic agents involved in the initiation and progression of pulmonary fibrosis (Tam et al., 2021). Elevated levels of TGFB1 were found in the lungs of STZ-induced diabetic rats and were associated with pulmonary fibrosis (Talakatta et al., 2018). However, in the MIDY lung, the abundance of TGFB1 was not increased, and CTGF was even reduced by 1.7-fold (Benjamini–Hochberg-corrected *P*-value=0.01). The absence of a pro-fibrotic environment in the MIDY lung might be related to the elevated levels of SLRPs, which modulate the expression and activity of TGFB1 and CTGF and could therefore potentially protect the tissue against their deleterious effects (Nastase et al., 2014). Furthermore, SLRP levels were correlated significantly and, together, SLRPs could counteract the vicious cycle observed previously in the diabetic lung, being characterized by elevated production of the pro-fibrotic growth factors and increased matrix deposition. In line with this, analysis of histological lung tissue sections from MIDY and WT pigs did not reveal evidence of fibrosis in the MIDY lung. The levels of different members of SLRPs were also elevated in other diabetic conditions such as human diabetic nephropathy (Schaefer et al., 2001), diabetic foot ulceration (Theocharidis et al., 2022), type 2 diabetes and obesity (Bolton et al., 2012). In the case-cohort study, decorin (DCN) – one of the best characterized SLRPs – was selected as one of the most important biomarkers for type 2 diabetes prediction (Thorand et al., 2021). Furthermore, the occurrence of sterile inflammation, characterized by a low-grade inflammatory response, is considered to contribute to pulmonary complications in hyperglycaemic conditions. Reduced complement system activity and humoral immunity associated with a reduced response of specialized immune cells increase the risk of infections in patients with diabetes (Muller et al., 2005). In line with this, gene set enrichment analysis of proteomics data from the MIDY lung revealed proteins related to the regulation of the humoral immune response to be the most overrepresented in the set of downregulated proteins (among others, serpin family A members, complement and coagulation proteins). In line with this, a proteomics study of human type 1 diabetes serum revealed dysregulation of proteins involved in innate immune responses and in the activation of the complement cascade (Zhang et al., 2013). Taken together, the humoral immune response appears to be compromised in the MIDY lung, potentially worsening the defence response.

A particularly novel and interesting finding of this investigation is a prominent, 2.5-fold downregulation of PUFA lipoxygenase ALOX15 in the MIDY lung. Alterations in ALOX15 regulation have been observed in various cardiovascular, renal, neurological and metabolic disorders (reviewed by Singh and Rao, 2019). Although the existence of ALOX15 orthologues has been known for several decades, their biological role is still under discussion. Like other lipoxygenases, ALOX15 is involved in the metabolism of PUFAs to form biologically active lipid mediators. The physiological substrates of ALOX15 are linoleic acid (LA), α-linolenic acid (ALA), γ-linolenic acid (GLA), arachidonic acid (AA), eicosapentaenoic acid (EPA) and docosahexaenoic acid (DHA). In the lung, ALOX15 products can stimulate or resolve inflammation and stimulate tissue repair (Abrial et al., 2015). A recent review highlighted the importance of ALOX15 in the formation of key lipid mediators to terminate inflammation during lung cancer in humans (Tian et al., 2017). The strong downregulation of ALOX15 in the MIDY lung appears to be indicative of a disturbed immune response. Besides, leukotriene A(4) hydrolase (LTA4H) was moderately elevated in the MIDY lung. LTA4H converts leukotriene A4 (LTA4) to leukotriene B4 (LTB4) and therefore plays an important role in the generation of pro-inflammatory leukotrienes. A shift from leukotriene to lipoxin production, also known as eicosanoid class switching, is necessary to resolve inflammation and to prevent the progression to chronic inflammation (Ringholz et al., 2014). The inverse regulation of LTA4H and ALOX15 therefore possibly indicates the unbalanced production of pro-inflammatory lipid mediators. This agrees with the observed dysregulation of proteins related to the humoral immune response in the MIDY lung discussed above. Furthermore, the lipidomics dataset showed a trend of lower levels of lipoxygenase products in the MIDY lung, which is concordant with the strongly reduced protein levels of ALOX15. Eicosanoid levels derived from the lipoxygenase pathway were strongly correlated, suggesting an orchestrated co-regulation of these molecules. The most pronounced changes in the levels of these molecules were downregulation of 14-hydroxydocosahexaenoic acid (14-HDHA) and 12-hydroxyeicosatetraenoic acid (12-HETE). 12-HETE, which can be produced by ALOX15, is known to have pro- and anti-inflammatory effects (Snodgrass and Brüne, 2019). 14-HDHA, which was reduced by ∼2.2-fold, is produced through the ALOX15-catalyzed oxygenation of DHA and is the key precursor of maresin, an anti-inflammatory lipid mediator (Snodgrass and Brüne, 2019). Taken together, strongly reduced ALOX15 and associated eicosanoid levels reflect imbalanced production of pro- and anti-inflammatory mediators in the MIDY lung and provide molecular insights into the impoverished ability of inflammation resolution as a hallmark of diabetic lung disease.

In conclusion, this is the first multi-omics characterization of lung tissue in a clinically relevant large-animal model of insulin-deficient diabetes mellitus. The fact that – for logistic reasons – only female pigs could be maintained for 2 years represents a limitation of this study. Another limitation of the study is the relatively small group size, which may explain why some of the findings are only trends close to the significance threshold. However, the combination of multiple layers of molecular information with rigorous statistical and bioinformatic approaches revealed previously unreported functional consequences of insulin deficiency for the lungs. To rule out the possibility that the proteome differences found between MIDY and WT samples could be due to different levels of blood contamination, we compared the concentrations of the of haemoglobin subunit β (HBB) in the samples (Fig. S4). The fact that these did not differ between the two groups argues against a systematic bias of the results by different levels of blood contamination. The generated datasets further provide an important resource for future studies on the progression of pulmonary complications and other associated comorbidities in diabetes mellitus. In particular, it will be interesting to see if the molecular alterations observed in lung tissue of MIDY pigs are reflected in samples from patients with severe insulin-deficient diabetes or other forms of diabetes. Furthermore, MIDY pigs provide an interesting model for testing if diabetes treatments, e.g. insulin replacement therapies or SGLT2 (also known as SLC5A2) inhibitors, can revert the observed pulmonary alterations.

## MATERIALS AND METHODS

### Biological samples

Samples were taken from female German Landrace-Swabian-Hall crossbred pigs. Female MIDY pigs (hemizygous *INS*^C94Y^ transgenic; *n*=4) were maintained with suboptimal insulin treatment for 2 years (fasting plasma glucose levels >250 mg/dl), together with female WT littermates (*n*=5). At the age of 2 years, all pigs were sacrificed for generation of an extensive biobank collection (the ‘Munich MIDY Pig Biobank’) of representative tissue samples from a broad spectrum of different organs and tissues (Blutke et al., 2017). Overnight fasted pigs were anesthetized by intramuscular injection of ketamine (Ursotamin^®^, Serumwerk Bernburg) and azaperone (Stresnil^®^, Elanco Animal Health), followed by intravenous application of ketamine and xylazine (2% Xylazin, Serumwerk Bernburg). Animals were then euthanized under anaesthesia by intravenous injection of T61^®^ (Intervet) and immediately subjected to necropsy. WT and MIDY pigs were euthanized in alternate order. After death, the carcasses were suspended at the hind legs and the head was dissected to achieve maximal exsanguination. Necropsy, collection and processing of representative tissue samples were performed according to established standard sampling protocols for porcine biomedical models (Albl et al., 2016). Representative samples of fresh lung tissue were systematically randomly sampled, excised, subdivided and differentially processed for different downstream analyses (Blutke et al., 2017). For molecular profiling (proteomic) analyses, tissue samples were shock frozen on dry ice and then stored at −80°C until further investigation. For histology, immunohistochemistry and quantitative histomorphological analyses, tissue samples were routinely processed for paraffin histology (Blutke et al., 2017). All experiments were performed according to the German Animal Welfare Act and approved by the Government of Upper Bavaria, following the ARRIVE guidelines and Directive 2010/63/EU for animal experiments.

### Proteomics

#### Sample preparation

Frozen lung tissue samples were washed briefly in ice-cold phosphate-buffered saline (PBS) supplemented with protease inhibitors (Roche Diagnostics, Mannheim, Germany). Samples were snap frozen in liquid nitrogen and transferred into prechilled tubes and cryo-pulverized in a CP02 Automated Dry Pulverizer (Covaris, Woburn, MA, USA) using an impact level of five according to the manufacturer's instructions. Powdered tissue was lysed in 8 M urea/0.5 M NH_4_HCO_3_ supplemented with protease inhibitors (Roche Diagnostics) by ultrasonication (18 cycles of 10 s) using a Sonopuls HD3200 (Bandelin, Berlin, Germany). Pierce 660 nm Protein Assay (Thermo Fisher Scientific, Rockford, IL, USA) was used for protein quantification. 20 µl of lysate containing 50 µg of protein was processed for digestion. Briefly, disulfide bonds were reduced [45 mM dithiothreitol/20 mM tris(2-carboxyethyl) phosphine, 30 min, 56°C] and cysteine residues were alkylated (100 mM iodoacetamide, 30 min, room temperature), followed by quenching of excess iodoacetamide with dithiothreitol (90 mM, 15 min, room temperature). Proteins were then digested sequentially, firstly with Lys-C (FUJIFILM Wako Chemicals Europe GmbH, Neuss, Germany) for 4 h (1:50 enzyme to protein ratio) and subsequently with modified porcine trypsin (Promega, Madison, WI, USA) for 16 h at 37°C (1:50 enzyme to protein ratio). Peptides were then desalted using a Sep Pak C18 cartridge (Waters, Milford, MA, USA) according to the manufacturer's instructions. The SepPak eluents were dried before analysis using a vacuum centrifuge.

#### Nano-LC-MS/MS analysis

1 μg of the digest was injected on an UltiMate 3000 nano-LC system coupled online to a Q-Exactive HF-X instrument (Thermo Fisher Scientific) operated in the DIA mode. Peptides were transferred to a PepMap 100 C18 trap column (100 µm×2 cm, 5 µM particles, Thermo Fisher Scientific) and separated on an analytical column (PepMap RSLC C18, 75 µm×50 cm, 2 µm particles, Thermo Fisher Scientific) at 250 nl/min with an 80-min gradient of 5-20% of solvent B, followed by a 9-min increase to 40% solvent B. After the gradient, the column was washed with 85% solvent B for 9 min, followed by a 10-min re-equilibration with 3% solvent B. Solvent A consisted of 0.1% formic acid in water and solvent B of 0.1% formic acid in acetonitrile. The Q-Exactive HF-X instrument was configured to acquire 50×12 m/z-wide (in the range of 400-1000 m/z) precursor isolation window DIA spectra [15,000 resolution; automatic gain control (AGC) target, 1×10^6^; maximum ion injection time (IIT), 20 ms; nominal collision energy (NCE), 27%] as described previously (Pino et al., 2020; Shashikadze et al., 2023) using a staggered window pattern (Amodei et al., 2019) with window placements optimized by Skyline software (v. 21.1) (MacLean et al., 2010). Precursor spectra (in the range of 390-1010 m/z; 60,000 resolution; AGC target, 1×10^6^; maximum IIT, 60 ms; +3H assumed charge state) were interspersed among every 50 ms/ms spectra. Chromatogram libraries using gas-phase fractionation (Searle et al., 2018) were built using the same LC settings. Six injections of pooled digest were performed with 25×4 m/z-wide DIA (30,000 resolution; AGC target, 1×10^6^; maximum IIT, 55 ms; NCE, 27%; +3H assumed charge state) using a staggered window pattern with window placements optimized by Skyline software (v. 21.1) (i.e. 400.43-502.48, 500.48-602.52, 600.52-702.57, 700.57-802.61, 800.61-902.66 and 900.66-1002.70), producing 300×2 m/z-wide windows spanning from 400 to 1000 m/z after deconvolution. Table S9 contains the actual windowing schemes.

#### Identification, quantification and bioinformatics

Raw data processing was carried out using DIA-NN (v1.8) (Demichev et al., 2020). Identification was based on predicted spectral libraries generated by the built-in deep-learning-based spectra and retention time predictor in DIA-NN and further constrained by experimental data from project-specific gas-phase fractionation-based libraries (also generated by DIA-NN). For all searches, the *Sus scrofa* protein database (UniProt Reference Proteome, taxonomy 9823, proteome ID UP000008227, last modified 16 June 2021, 49,792 entries) alongside the MaxQuant contaminants fasta file (Tyanova et al., 2016) were used. The enzyme for digestion was set to trypsin and one missed cleavage was allowed. Only peptides with a charge state of +2, +3 and +4 were considered. Cysteine carbamidomethylation was set as a fixed modification. The precursors were filtered at 1% false discovery rate. Retention time correction was performed automatically by DIA-NN and the quantification strategy was set to ‘Robust LC’ (high accuracy mode). Similarly, mass tolerance was determined automatically by DIA-NN as described previously (Demichev et al., 2020) and was set to 8 ppm and 20 ppm for MS1 and MS2, respectively. The top six fragments (based on their reference library intensities) were used to calculate raw intensities for precursors. The ‘Genes’ column was used to count unique proteins (as gene products were identified and quantified using proteotypic peptides only). All other settings were left as default. Table S10 contains detailed description of DIA-NN parameters used in this study. The main output containing precursor level data from DIA-NN was used for the downstream analysis in R (https://www.r-project.org/) using custom scripts. Briefly, the output was filtered at 1% false discovery rate, using experiment-wide q-values for protein groups and both experiment-wide and run-specific q-values for precursors. Non-proteotypic peptides, peptides with a low signal quality and peptides derived from potential contaminants were excluded from further analysis. Precursor intensities for different charge states were aggregated to the peptide level by taking the sum of intensities. Peptide intensities were normalized and proteins with at least two unique peptides detected in at least three biological replicates of each condition were tested for differential abundance using the MS-EmpiRe algorithm (Ammar et al., 2019). The STRING-pre-ranked gene set enrichment analysis (Szklarczyk et al., 2019) was used to reveal biological pathways associated with differentially abundant proteins between MIDY and WT samples. Signed log-transformed *P*-values were used as ranking metrics and the false discovery rate was controlled at 5%. To minimize redundancy, significant Gene Ontology (GO) biological processes were grouped into similar ontological terms with REVIGO (Supek et al., 2011) at an allowed similarity of 0.7.

### Targeted lipidomics

#### Sample preparation for analysis of PUFA-derived lipid mediators and metabolites

An antioxidant cocktail consisting of 0.2 mg/ml butylated hydroxytoluene (CAS 128-37-0; Merck, Darmstadt, Germany), 100 µM indomethacin (CAS 53-86-1; Merck) and 100 µM TPPU (CAS 1222780-33-7; Merck) was added to 10-30 mg of the thawed tissue sample to protect the sample from oxidation during sample preparation. Additionally, a deuterated internal standard mix consisting of 14,15-DHET-d_11_, 15-HETE-d_8_, 20-HETE-d_6_, 8,9, EET-d_11_, 9,10-DiHOME-d_4_, 12(13)-EpOME-d_4_, 13-HODE-d_4_, PGB2-d_4_ and LTB4-d_4_ (100 pg each; Cayman Chemical, Ann Arbor, USA) was spiked in. Methanol and sodium hydroxide were added for protein precipitation and alkaline hydrolysis at 60°C for 30 min. After solid-phase extraction, the eluate was evaporated (Rivera et al., 2004) to obtain a solid residue which was dissolved in 100 µl methanol/water (60:40 v/v). The residues were analysed using an Agilent 1290 HPLC system with binary pump, multi-sampler and column thermostat with a Zorbax Eclipse plus C-18, 2.1×150 mm, 1.8 µm column using a gradient solvent system of aqueous acetic acid (0.05%) and 50:50 acetonitrile/methanol. The flow rate was set at 0.3 ml/min and the injection volume was 20 µl. The HPLC was coupled with an Agilent 6495 triple quadrupole mass spectrometer (Agilent Technologies, Santa Clara, USA) with an electrospray ionization source. Analysis was performed with multiple reaction monitoring (MRM) in negative mode with at least two mass transitions for each compound. All oxylipins were individually calibrated using authentic standards purchased from Cayman Chemical in relation to the deuterated standard. Certified MaxSpec^®^ quality was used if available. If not, the uncertified standards have been adapted to MaxSpec^®^ standards of similar compounds.

#### Sample preparation for analysis of PUFAs

All compounds were purchased from Cayman Chemicals.

#### Preparation of tissue samples and quality controls

Porcine lung tissue samples were weighted into homogenization tubes with ceramic beads (1.4 mm) (Bertin P000933-LYSK0A tubes). To each 1 mg of frozen porcine lung tissue, 3 μl of a cooled mixture (4°C) of ethanol/phosphate buffer (85:15, v/v) was added. Tissue samples were homogenized using a Precellys^®^ 24 homogenizer (PEQLAB Biotechnology GmbH, Germany) three times for 30 s at 5500 rpm and 4°C, with 30 s pause intervals to ensure constant temperature. 30 µl (equivalent to 10 mg) of the lung homogenates were transferred into a 1.5 ml Eppendorf tube. Quality-control pool samples were prepared in triplicates by taking out 20 µl from each study sample. The pool sample was subsequently mixed and 30 µl was transferred into 1.5 ml Eppendorf tubes.

Quality-control reference samples were prepared in triplicates in 1.5 ml Eppendorf tubes by mixing 5 µl of the standard mixture consisting of AA, ALA, LA, dihomo-γ-linolenic acid (DGLA), DHA and EPA (300 ng/ml) with 45 µl of water. Blank (triplicate) and zero (single) samples were prepared by transferring 30 µl of ethanol/phosphate buffer (85:15, v/v) into 1.5 ml Eppendorf tubes. Calibrators were prepared in 1.5 ml Eppendorf tubes by successive dilutions (factor 3) in water/methanol (50:50, v/v) of the calibration mixture consisting of AA, ALA, LA, DGLA, DHA and EPA (2000 ng/ml) to reach nine calibrator points (cal.): 666.67 ng/ml (cal. 09) to 0.102 ng/ml (cal. 01). 30 µl of each calibrator point was then transferred to a new 1.5 ml Eppendorf tube.

Every tube was pre-cooled in wet ice before starting sample preparation and kept on wet ice all along the extraction procedure.

For accurate quantification, 10 µl of the internal standard mixture consisting of AA-d_5_, DHA-d_5_ and EPA-d_5_ (ISTD mixture) (50 ng/ml) was added to the samples, except the zero sample.

#### Extraction procedure

For lipid extraction, 150 µl of cold methanol (−20°C) was added to the samples, followed by incubation for 10 min with vortexing every 3 min. Protein precipitation was performed by centrifugation of the samples at 10,000 ***g*** for 15 min at 4°C. The supernatant (around 150 µl) was transferred to a 1 ml Nunc 96-well polypropylene plate (Thermo Fisher Scientific), and the volume was adjusted with water to reach 1 ml (final methanol concentration of 15%) and mixed. Solid-phase extraction was then performed with a Strata-X Micro 96-well plate, 33 µm, 2 ml (Phenomenex), using a positive pressure-96 processor (Waters). After solid-phase extraction plate conditioning with two 0.5 ml methanol washes and then two 0.5 ml water washes, 2×0.5 ml of each sample were loaded on the SPE plate. After rinsing twice with 0.5 ml 10% methanol in water (v/v), the analytes were eluted twice with 100 µl methanol into a new 1 ml 96-well plate. Samples were transferred to a select-a-vial 96-well plate with 300 µl glass inserts (Analytical Services) and evaporated to dryness at 30°C with nitrogen gas. Analytes were resuspended with 30 µl 50% methanol in water (v/v), vortexed, and centrifuged for a few seconds at 1000 ***g*** before direct injection into the analytical system.

#### LC-MS/MS analysis

All samples were measured with an Exion UHPLC-system coupled to a QTRAP 6500+ mass spectrometer (SCIEX, Darmstadt, Germany) operated with Analyst 1.6.3. Chromatographic separation was achieved using a Kinetex C18 reversed phase column (1.7 μm, 100×2.1 mm, Phenomenex) with a SecurityGuard Ultra Cartridge C18 (Phenomenex) precolumn, heated at 40°C. Mobile phases A [water:acetonitrile (70:30, v/v)+100 µl acetic acid (Honeywell Fluka, 15655660)] and B [acetonitrile/isopropanol (50:50, v/v)] were used in a gradient program with an isocratic flow rate of 500 µl/min as follows: 0% B at 0 min, 70% B at 6.5 min, 100% B at 7.8 min, 100% B at 9.5 min, and 0% B at 11 min. The autosampler was operated at 4°C with an injection volume of 10 µl of sample.

The coupled mass spectrometer was equipped with an electrospray ionization Turbo-VTM source set to negative mode. Source parameters were optimized to the following values: source temperature, 500°C; curtain gas flow, 40 psi; ion spray voltage, −4000 V; ion source gas 1, 50 psi; ion source gas 2, 40 psi. Metabolites were analysed via scheduled MRM (sMRM) with nitrogen as the collision gas. All MRM transitions were optimized for each compound, as well as the source parameters such as declustering potential, collision energy, cell exit potential and entrance potential. The sMRM detection window was set to 60 s. Acquisition time was about 8.5 min.

SciexOS software v2.2.0.5738 (SCIEX) was finally used for peak detection, integration and quantitation of compounds (MQ4 algorithm). For quantification, a weighted linear regression was calculated from extracted calibrator samples for every compound using the area ratio between the analyte and its internal standard. The values on the *x*-axis (ratio of the actual analyte concentration and the actual calibrator concentration) were weighted by 1/x.

### Bioinformatics

PCA was performed to discover natural grouping existing in the data. PCA was built on log-transformed data using the prcomp function from the R package ‘stats’ (https://www.R-project.org/). To reveal eicosanoid subclasses with a similar regulation pattern, correlation analysis with rank-based approach (Spearman correlation) was used. The significance of correlation (*P*-value) was corrected for all pairwise comparisons with the Benjamini–Hochberg procedure using the R package ‘psych’ (https://cran.r-project.org/package=psych). The correlation matrix was first subjected to hierarchical clustering using complete linkage clustering as the clustering method and the Spearman correlation as the distance measure (Gu et al., 2016). The resulting heatmap was partitioned into four different clusters using the k-means algorithm. A correlation matrix was also visualized as a network using the R package ‘igraph’ (https://igraph.org/). Community detection was performed using the walktrap algorithm, which attempts to find densely populated subnetworks by random walks (Pons and Latapy, 2006). Focusing on similarities between proteomics and lipidomics data, CIA was performed using the R package ‘omicade4’ (Meng et al., 2014) to assess global measures for the co-variability of two datasets. The similarity between the two datasets was evaluated with the parameter RV, which is a multivariate extension of the Pearson correlation coefficient. The significance of the RV coefficient was assessed with a permutation test consisting of 500 iterations.

### Histopathology, immunohistochemistry and quantitative morphological analyses

For qualitative and quantitative histomorphological analyses, paraffin sections stained with Haematoxylin and Eosin or Masson's trichrome stain (connective tissue stain) were examined. Immunohistochemical detection of ALOX15 was performed using the following antibodies: mouse monoclonal anti-ALOX15 (clone OTI7H6, #TATA504358, Origene, 1:150), followed by biotinylated goat-anti-mouse secondary antibody (#115-065-146, Jackson ImmunoResearch, 1:500) and horseradish peroxidase-labelled avidin-biotin complex (#PK-6100, Vector Laboratories). Immunoreactivity was visualized using 3,3′-diaminobenzidine tetrahydrochloride dihydrate (DAB). Sections stained with buffer instead of the primary antibody were used as the negative control. The volume density of ALOX15-positively labelled cells within the lung [V_V(ALOX15-positive cells/lung)_] was determined following the principle of Delesse (Howard and Reed, 2004) and calculated as the sum of cross-sectional areas of ALOX15-positive cell profiles, divided by the sum of cross-sectional areas of lung tissue (excluding air-filled spaces) in 48±2 systematically randomly sampled section areas per case. ALOX15-positive area densities were determined by differential point counting, using an automated stereology system (VIS-Visiopharm Integrator System v3.4.1.0 with newCAST software, Visiopharm A/S, Denmark), as previously described (Backman et al., 2019; Howard and Reed, 2004). In each case, >100,000 points were counted. The volume density of interstitial connective tissue within the lung [V_V(interstitial connective tissue/lung)_], was determined analogously in Masson trichrome-stained lung tissue sections (counting >10,000 points per case). All quantitative morphological analyses were performed in a masked manner, i.e. without knowing the affiliation of the examined animals. Statistical significance of the difference in the volume density of ALOX15-positively labelled cells and volume density of interstitial connective tissue in the lung between MIDY and WT samples were evaluated using two-sample Mann–Whitney *U*-test.

### Statistical analysis

During analysis, all samples were processed in parallel to avoid possible bias related to different storage times. Histology and immunohistochemistry were performed on lung tissue samples taken from exactly the same locations as the proteomic and lipidomic analysis samples. All statistical analyses were performed in R. Samples were analysed with a DIA method with MS1 spectra interspersed every 50 ms/ms scans. Identification was performed using DIA-NN (Demichev et al., 2020) and its built-in deep learning-based spectra and retention time predictor alongside project-specific narrow-window gas-phase fractionation-based library. A false discovery ate cut-off of 1% was applied on precursor and protein levels. The MS-EmpiRe workflow (Ammar et al., 2019) followed by a Benjamini–Hochberg multiple testing correction was used to reveal differentially abundant proteins. Correlation between selected variables was evaluated using Spearman correlation and the resulting *P*-values were corrected for all pairwise comparisons using the Benjamini–Hochberg method.

## Supplementary Material

10.1242/dmm.050650_sup1 Supplementary information

Table S1. Peptides identified and quantified by nano-LC-MS/MS-based DIA proteomics.

Table S2. Protein groups identified by nano-LC-MS/MS-based DIA proteomics.

Table S3. Results of MS-EmpiRe-based quantitative proteomics of MIDY vs. WT pigs. Proteins with BH adjusted p-value < 0.05 are shown. Positive log2 fold change means more abundant in the MIDY group

Table S4. STRING Functional Enrichment Analysis of MIDY vs. WT. Gene Ontology (GO) biological processes with direction “top” are enriched for proteins less abundant and with direction “bottom” are enriched for proteins more abundant in MIDY vs. WT. Processes with “both ends” are simualtanously enriched for proteins more and less abundant

Table S5. Results of quantitative targeted lipidomics of MIDY vs. WT pig (shown as ng/g tissue).

Table S6. Global correlation matrix of quantified eicosanoid levels. The correlations were estimated using the non-parametric Spearman correlation method. Color gradient corresponds to the magnitude of the correlation

Table S7. Statistical analysis of targeted lipidomics data. Positive log2 fold changes means more abundant in the MIDY group. P-values are calculated using two-tailed Welch's t-test. Variance importance in projection (VIP) scores are from the orthogonal partial least squares discriminant analysis (OPLS-DA) model

Table S8. Results of quantitative targeted lipidomics of selected eicosanoid precursors from MIDY vs. WT pigs (shown as ng/g tissue).

Table S9. Window placements optimized by Skyline software (v.21.1) for the single-injection DIA runs and gas-phase fractionation (GPF) DIA runs.

Table S10. Detailed description of DIA-NN parameters.
