# Supplementary material for: Multi-omics analysis of diabetic pig lungs reveals molecular derangements underlying pulmonary complications of diabetes mellitus
Source: Dis Model Mech. 2024 Jul 23;17(7):dmm050650. doi: 10.1242/dmm.050650 (PMC11583917; doi:10.1242/dmm.050650)
Supplement: Supplementary information [file dmm-17-050650-s1.pdf]

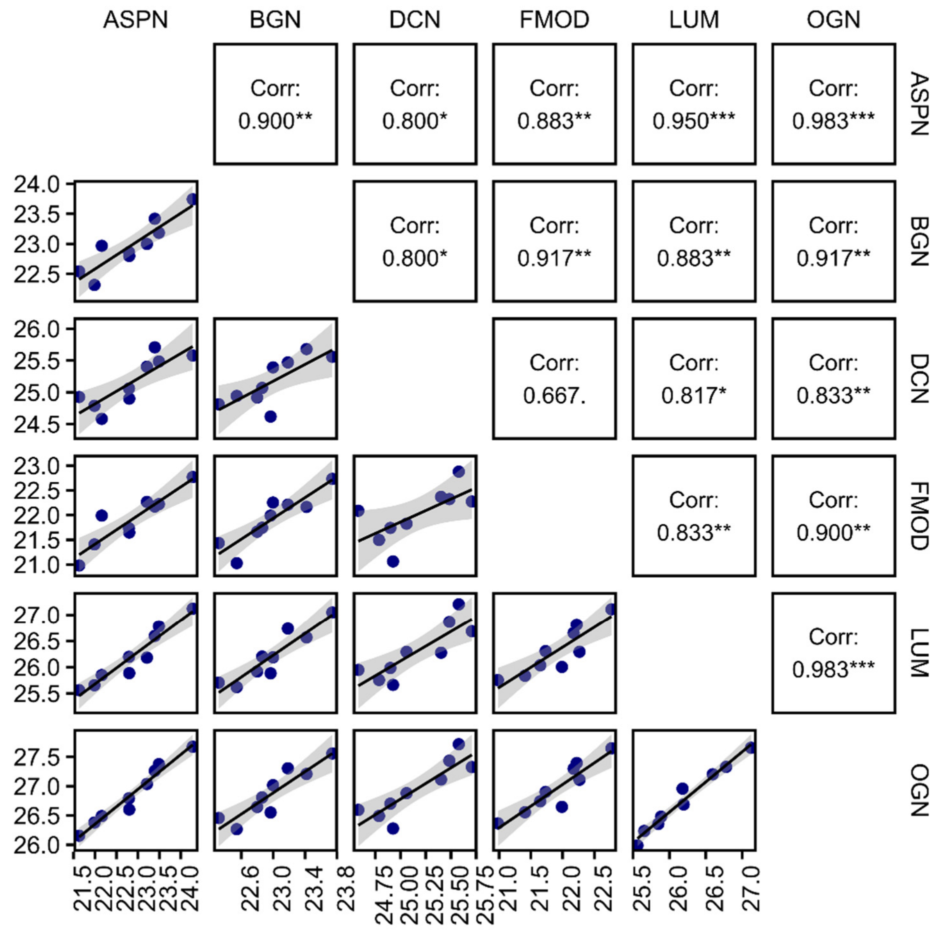

Fig. S1. Multi-scatter plot of small-leucine rich proteoglycan levels in all animals with regression line (black solid line) and confidence interval (grey area) (lower triangle). Spearman correlation coefficient and the significance of the correlation (p-value) is shown in the upper triangle. *p < 0.05; **p < 0.01; ***p < 0.001

Disease Models & Mechanisms • Supplementary information

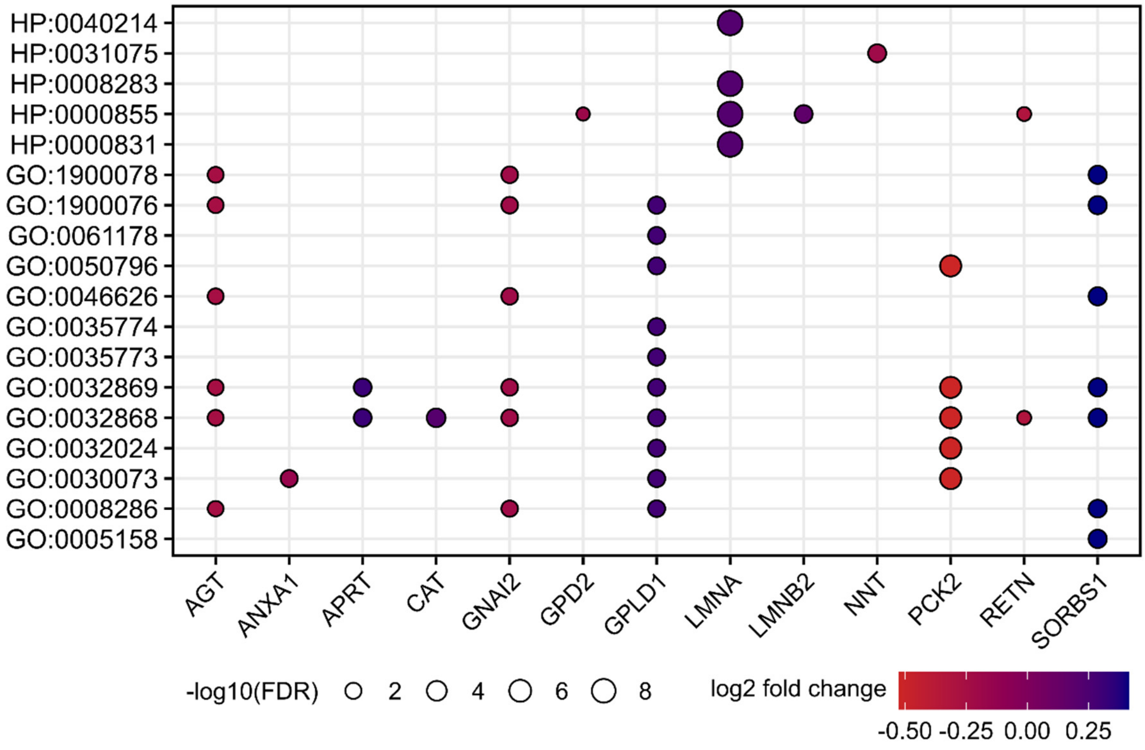

Fig. S2. Abundance change of proteins (adjusted p-value < 0.05) related to HP:0040214 (abnormal insulin level), HP:0031075 (abnormal response to insulin tolerance test), HP:0008283 (fasting hyperinsulinemia), HP:0000855 (insulin resistance), HP:0000831 (insulin resistant diabetes mellitus), GO:1900078 (positive regulation of cellular response to insulin stimulus), GO:1900076 (regulation of cellular response to insulin stimulus), GO:0061178 (regulation of insulin secretion involved in cellular response to glucose stimulus), GO:0050796 (regulation of insulin secretion), GO:0046626 (regulation of insulin receptor signaling pathway), GO:0035774 (positive regulation of insulin secretion involved in cellular response to glucose stimulus), GO:0035773 (insulin secretion involved in cellular response to glucose stimulus), GO:0032869 (cellular response to insulin stimulus), GO:0032868 (response to insulin), GO:0032024 (positive regulation of insulin secretion), GO:0030073 (insulin secretion), GO:0008286 (insulin receptor signaling pathway), GO:0005158 (insulin receptor binding). The color of the bubble corresponds to the log2 fold change of protein (red downregulation, blue upregulation) and the size of the bubble indicates the significance of the protein change. AGT, angiotensin 1-10; ANXA1, annexin; APRT, adenine phosphoribosyltransferase; CAT, catalase; GNAI2, G protein subunit alpha i2; GPD2, glycerol-3-phosphate dehydrogenase; GPLD1, glycosyl-phosphatidylinositol-specific phospholipase D; LMNA, prelamin-A/C; LMNB2, lamin B2; NNT, proton-translocating NAD(P)(+) transhydrogenase; PCK2, phosphoenolpyruvate carboxykinase (GTP); RETN, resistin; SORBS1, sorbin and SH3 domain containing 1.

Disease Models & Mechanisms • Supplementary information

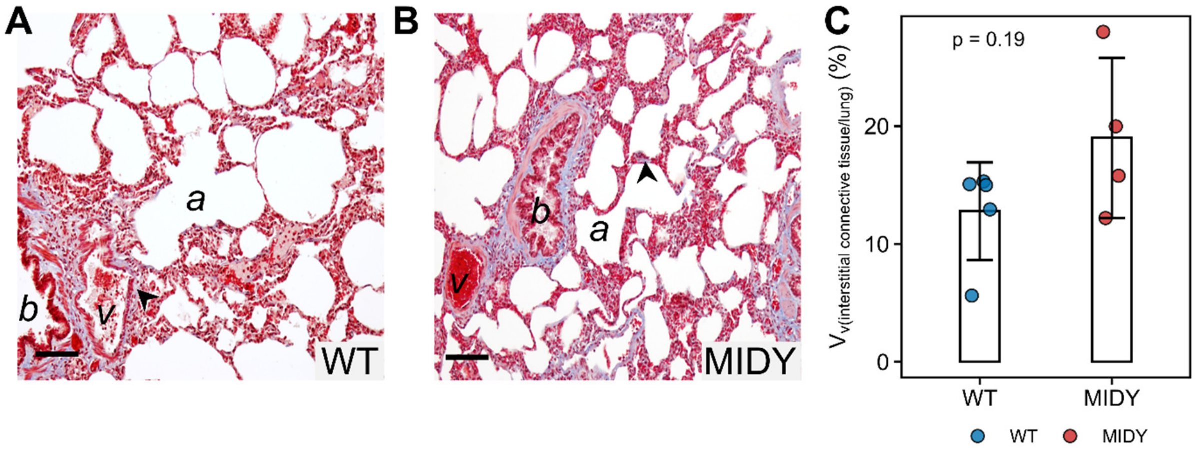

Fig. S3. Detection and quantification of interstitial connective tissue in the WT and MIDY lung. Masson's trichrome-stained lung sections of MIDY (A) and WT (B) pigs. Histological landmarks (alveoli (a), blood vessels (v), and bronchioli (b)) are indicated. Connective tissue stains blue. Size bars: 100 µm. (C) Volume densities (in %) of interstitial connective tissue in the lung (excluding air-filled alveolar spaces). Statistical significance was assessed by the two-sample Mann–Whitney U Test. The bar diagrams show means and standard deviations.

A Hematological parameters in MIDY and WT pigs

ID Pig	Genotype	Erythrocytes (T/l)	Hemoglobin (g/l)	Hämatokrit (l/l)	MCHC (g/l)
1857	MIDY	4.97	110	0.31	361
1856	MIDY	5.02	107	0.30	351
1859	MIDY	4.53	106	0.28	376
1886	MIDY	4.61	110	0.30	370
1861	WT	5.23	107	0.30	356
1885	WT	3.54	81	0.23	351
1877	WT	4.89	103	0.28	373
1875	WT	4.37	105	0.27	381
1878	WT	4.74	106	0.29	370
	MIDY Mean	4.78	108.25	0.30	364.50
	MIDY SD	0.25	2.06	0.01	10.91
	WT Mean	4.55	100.40	0.27	366.20
	WT SD	0.65	10.95	0.03	12.40
	T-Test p =	0.4976	0.1859	0.1362	0.8333

B Quantification of hemoglobin subunit beta In lung tissue

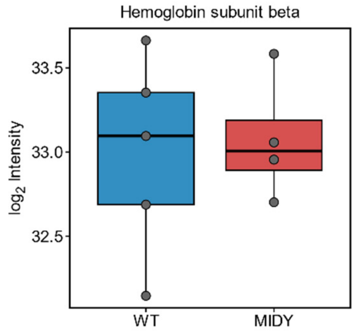

Fig. S4. Hematological parameters and quantification of hemoglobin in lung tissue of MIDY and WT pigs. A) MIDY and WT pigs did not significantly differ in any of the hematological parameters investigated (from Blutke et al., Mol Metab. 2017 Jun 13;6(8):931-940. doi: 10.1016/j.molmet.2017.06.004. **B)** Quantification of hemoglobin subunit beta in lung tissue samples from MIDY and WT pigs by mass spectrometry shows variation (as expected), but no significant difference between the two groups, strongly arguing against a systematic bias by different levels of blood contamination of samples from MIDY and WT pigs.

Table S1. Peptides identified and quantified by nano-LC-MS/MS-based DIA proteomics.

Available for download at
https://journals.biologists.com/dmm/article-lookup/doi/10.1242/dmm.050650#supplementary-data

Table S2. Protein groups identified by nano-LC-MS/MS-based DIA proteomics.

Available for download at

https://journals.biologists.com/dmm/article-lookup/doi/10.1242/dmm.050650#supplementary-data

Table S3. Results of MS-EmpiRe-based quantitative proteomics of MIDY vs. WT pigs. Proteins with BH adjusted p-value < 0.05 are shown. Positive log2 fold change means more abundant in the MIDY group

Available for download at
https://journals.biologists.com/dmm/article-lookup/doi/10.1242/dmm.050650#supplementary-data

Disease Models & Mechanisms • Supplementary information

Table S4. STRING Functional Enrichment Analysis of MIDY vs. WT. Gene Ontology (GO) biological processes with direction "top" are enriched for proteins less abundant and with direction "bottom" are enriched for proteins more abundant in MIDY vs. WT. Processes with "both ends" are simualtanously enriched for proteins more and less abundant

Available for download at
https://journals.biologists.com/dmm/article-lookup/doi/10.1242/dmm.050650#supplementary-data

Table S5. Results of quantitative targeted lipidomics of MIDY vs. WT pig (shown as ng/g tissue).

Available for download at
https://journals.biologists.com/dmm/article-lookup/doi/10.1242/dmm.050650#supplementary-data

Table S6. Global correlation matrix of quantified eicosanoid levels. The correlations were estimated using the non-parametric Spearman correlation method. Color gradient corresponds to the magnitude of the correlation

Available for download at
https://journals.biologists.com/dmm/article-lookup/doi/10.1242/dmm.050650#supplementary-data

Table S7. Statistical analysis of targeted lipidomics data. Positive log2 fold changes means more abundant in the MIDY group. P-values are calculated using two-tailed Welch's t-test. Variance importance in projection (VIP) scores are from the orthogonal partial least squares discriminant analysis (OPLS-DA) model

Available for download at
https://journals.biologists.com/dmm/article-lookup/doi/10.1242/dmm.050650#supplementary-data

Table S8. Results of quantitative targeted lipidomics of selected eicosanoid precursors from MIDY vs. WT pigs (shown as ng/g tissue).

Available for download at
https://journals.biologists.com/dmm/article-lookup/doi/10.1242/dmm.050650#supplementary-data

Table S9. Window placements optimized by Skyline software (v.21.1) for the single-injection DIA runs and gas-phase fractionation (GPF) DIA runs.

Available for download at
https://journals.biologists.com/dmm/article-lookup/doi/10.1242/dmm.050650#supplementary-data

Table S10. Detailed description of DIA-NN parameters.

Available for download at

https://journals.biologists.com/dmm/article-lookup/doi/10.1242/dmm.050650#supplementary-data
